# Effect of attention on chewing and swallowing behaviors in healthy humans

**DOI:** 10.1038/s41598-019-42422-4

**Published:** 2019-04-12

**Authors:** Hirokazu Ashiga, Eri Takei, Jin Magara, Ryosuke Takeishi, Takanori Tsujimura, Kouta Nagoya, Makoto Inoue

**Affiliations:** 1 Division of Dysphagia Rehabilitation, Niigata University Graduate School of Medical and Dental Sciences, 2-5274 Gakkocho-dori, Chuo-ku, Niigata 951-8514 Japan; 2 Speech-Language and Hearing Therapy Course, Department of Rehabilitation, Faculty of Allied Health Sciences, University of Niigata Rehabilitation, 2-16 Kaminoyama, Murakami, 958-0053 Japan

## Abstract

We examined how attention alters chewing and swallowing behaviors. Twenty-one healthy volunteers were asked to freely eat 8 g of steamed rice in three separate trials, and we obtained the average number of chewing cycles (N) and chewing duration (T) prior to the first swallow in each trial. We also conducted an N-limited test, in which participants chewed the food while independently counting the number of chewing cycles and swallowed the food when they reached N, and a T-limited test, in which they chewed the food for T sec and then swallowed. We recorded electromyograms (EMGs) from masseter and suprahyoid muscles and collected videoendoscopic images. In the N-limited test, chewing speed decreased, masseter muscle activity (area under the curve of the rectified EMG burst) per cycle increased, and suprahyoid muscle activity per cycle decreased. In the T-limited test, the chewing speed increased, muscle activities per cycle decreased, and the number of cycles increased. The occurrence frequency of bolus propulsion into the pharynx before swallowing was smaller in the N- and T-limited tests than in the free chewing test. Further, the whiteout time was longer in the T-limited test than in the free chewing test. Attentional chewing changes not only chewing but also swallowing behavior.

## Introduction

Chewing is an early component of eating in humans. The aim of chewing is to crush the food between the upper and lower molar teeth, mix the triturated food particles with saliva to form a bolus, and propel the food bolus into the pharynx for swallowing. The motor patterns underlying chewing are programed by the central pattern generator (CPG) in the brain stem, and cortical inputs into the CPG can trigger rhythmic jaw movements in the absence of a food bolus^1^. However, various types of sensory information from the oral cavity, such as mechanical and chemical inputs or bolus location may influence masticatory performance. Numerous studies have reported that masticatory movements are highly adaptable. In particular, food texture or rheology can modulate masticatory forces^2–6^, jaw movements^7,8^, the length of a chewing cycle^9,10^, and the number of cycles preceding the swallow^11^. In addition, proprioceptive information obtained from muscle activity may serve as the sensory basis for food texture perception^12,13^. It should be noted that even healthy humans exhibit a wide range of inter-individual variation in masticatory behaviors^14–16^. For example, our previous study showed that chewing duration, defined as the amount of time from when a participant started to chew solid food to their first swallow, largely depends on individuals^17^. However, we did not clarify whether this individual variation reflected differences in masticatory function.

Volition may also affect chewing behaviors, including bolus preparation in the mouth and pharynx. Palmer *et al*.^18^ demonstrated that when participants were given instructions regarding chewing and swallowing food (command condition), bolus transport in the oral and pharyngeal cavities was significantly delayed. This suggests that volition may alter swallowing initiation in terms of both the timing and location of the food bolus at the start of the swallow. Furuya *et al*.^19^ showed that conscious effort during chewing altered chewing behaviors, specifically, chewing duration increased and bolus transit time during pharyngeal swallowing decreased. They concluded that volitional chewing with conscious effort could alter bolus transport and swallowing, leading to easier swallowing. However, these authors reported that chewing efficacy was reduced during volitional chewing; the mean chewing duration was 25.0 sec with volition and 13.6 sec without volition. Given these findings, volition may modulate elements of chewing behavior such as the chewing cycle, muscle force, and location of the bolus during chewing. Yet, the way in which volition or attentional effort impacts chewing behaviors followed by swallowing is still unclear.

In this study, we sought to (1) clarify how attention alters feeding behaviors such as chewing rhythm, muscle activity, and bolus propulsion prior to swallowing initiation and (2) evaluate how attention affects swallowing behavior. To this end, we chose three experimental conditions: free chewing, number limited (N-limited) chewing, and time limited (T-limited) chewing. In the N-limited test, the subjects were asked to count the number of chewing cycles and determine the timing of swallowing initiation independently. In the T-limited test, the subjects were asked to chew for T sec and then swallow when they received a cue from the experimenter. We conducted a physiological examination of chewing and swallowing behaviors in healthy humans using electromyography (EMG) and videoendoscopic (VE) imaging. We hypothesized that attention would have a minimal impact on masticatory muscle activity but that it would decelerate the bolus flow prior to swallowing initiation, leading to reduced swallowing efficacy and increased swallowing-related muscle activity.

## Results

### The free chewing test

**Figure 1:**
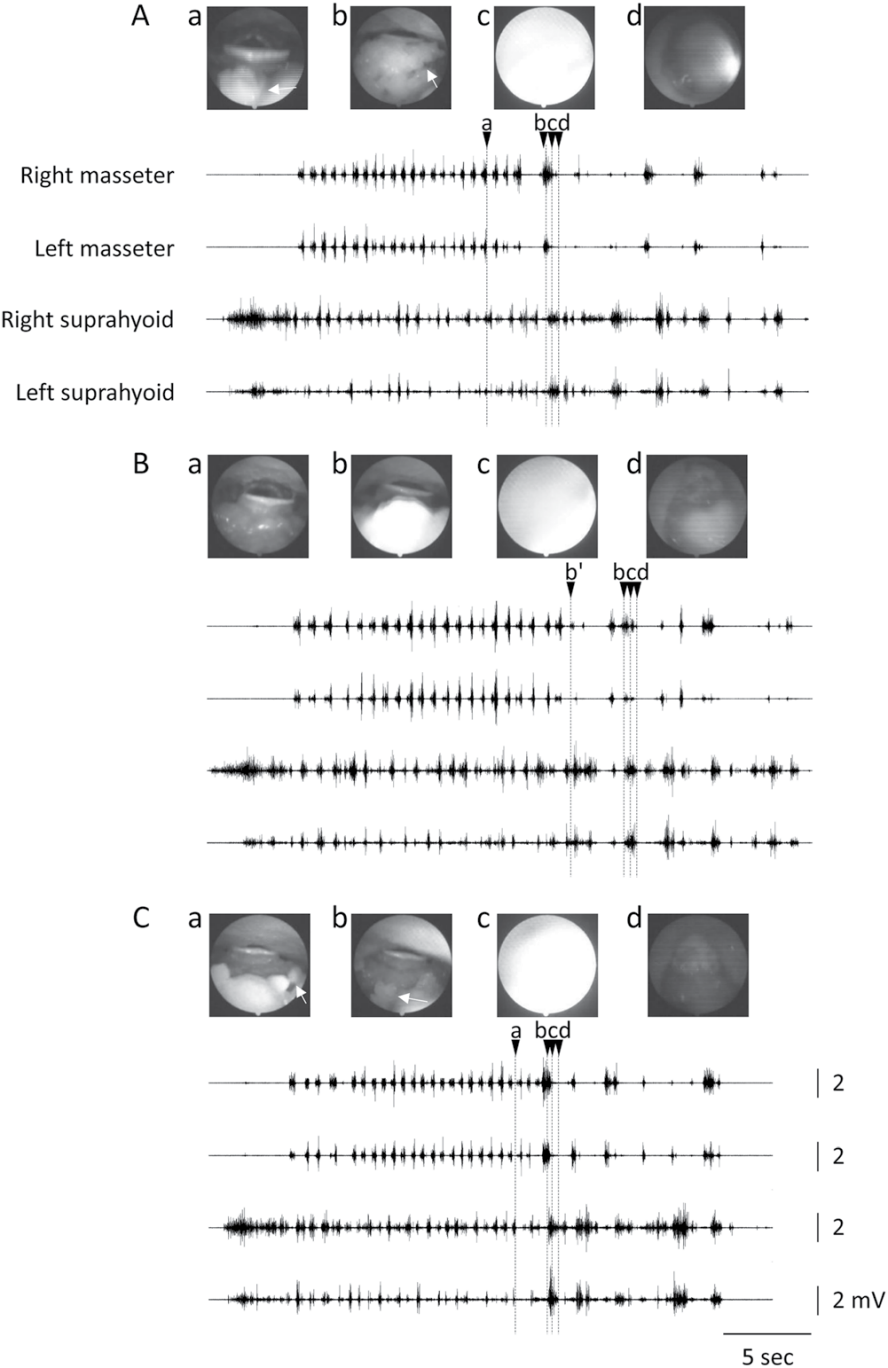
Simultaneous recording of EMGs and VE images. Representative recordings in the free (**A**), N-limited (**B**), and T-limited tests (**C**) are shown. (**A**) In this trial, the chewing duration was 12.50 sec and there were 22 chewing cycles. In this subject, the mean chewing duration and number of chewing cycles were 13.21 sec and 22 cycles, respectively. Top of (**A–C**) VE images for (a–d) above. Arrows in VE images indicate the food bolus. Vertical dotted lines a, b, c, and d represent the start of pharyngeal bolus propulsion, end of chewing (start of swallow), mid-part of pharyngeal swallowing (whiteout), and end of pharyngeal swallowing, respectively. Note that the end of chewing (b’) preceded the start of swallowing (b) in B.

All of the participants freely ate the test foods in a natural manner and did not report any discomfort. Typical recordings obtained during the free, N-limited, and T-limited tasks are shown in Fig. 1. The initial food intake appeared as a large burst of suprahyoid (Supra) EMG activity, followed by the start of chewing. Although the chewing side could not be determined from the masseter (Mas) and Supra EMG recordings alone, the rhythmic pattern of Mas EMG activity during chewing was stable in all cases, while the pattern of Supra EMG activity varied with each cycle. Swallowing was clearly distinguishable and occurred not only at the end of the sequence, but also between the chewing cycles.

First, we confirmed the reproducibility of the three free tasks using a one-way repeated measures analysis of variance or a one-way repeated measures analysis of variance on ranks followed by Tukey’s HSD test. Mas EMGs were not properly recorded in the third trial for one of the 21 participants, so we used the data obtained from the remaining 20 participants for the analysis. We found no significant difference in the mean value of chewing duration (15.7 ± 7.4 sec for the 1st, 14.5 ± 5.6 sec for the 2nd, and 15.9 ± 5.3 sec for the 3rd trial, n = 20, mean ± SD, *P* = 0.418), number of chewing cycles until the first swallow (24.7 ± 12.1 cycles for the 1st, 23.8 ± 10.6 cycles for the 2nd, and 26.0 ± 9.5 cycles for the 3rd trial, n = 20, mean ± SD, *P* = 0.285), swallow-related Supra EMG activity, defined as the area under the curve of the rectified EMG burst during swallowing (0.056 mV·sec [0.028–0.211 mV·sec] for the 1st, 0.065 mV·sec [0.027–0.184 mV·sec] for the 2nd, and 0.060 mV·sec [0.028–0.252 mV·sec] for the 3rd trial, n = 20, median [IQR25%–75%], P = 0.350), or whiteout time, which represents the pharyngeal swallowing time defined via the VE image (0.60 ± 0.08 sec for the 1st, 0.57 ± 0.07 sec for the 2nd, and 0.59 ± 0.07 sec for the 3rd trial, n = 20, mean ± SD, *P* = 0.106). These results indicate that our methods produced behavior that was reliably stable. We used averaged data obtained from the free chewing test for the following analysis.

Next, we examined sex differences in the basic behaviors. All participants seemed comfortable eating 8 g of rice. We found no significant differences in the mean value of chewing duration (15.2 ± 4.9 sec for men, n = 10 vs 15.3 ± 6.7 sec for women, n = 11, mean ± SD, *P* = 0.809), number of chewing cycles until the first swallow (25.8 ± 10.4 for men, n = 10 vs 23.1 ± 10.2 for women, n = 11, mean ± SD, *P* = 0.755), or whiteout time (0.59 ± 0.06 sec for men, n = 10 vs 0.57 ± 0.08 sec for women, n = 11, mean ± SD, *P* = 0.638).

### Effect of attention on chewing behaviors

We compared the characteristics of chewing performance among the conditions (Table 1). The number of chewing cycles was significantly greater in the T-limited test compared with the free chewing test. Chewing cycle time differed between the free chewing test and the N- and T-limited tests; N-limited > free > T-limited. Mas activity, defined as the area under the curve of the rectified EMG burst, was significantly larger in the N-limited test compared with the free chewing test, while we found no significant differences in Supra activity among the conditions. The Mas activity per chewing cycle in the free chewing test was significantly smaller than in the N-limited test and was significantly larger than that in the T-limited test. The Supra activity per chewing cycle was significantly larger in the free chewing test than in the N and T-limited test. These results show that attentional chewing may modulate the chewing speed and number of chewing cycles prior to swallowing as well as Mas and Supra activity. In the N-limited condition, although the subject determined the timing of swallowing, Mas activity slightly but significantly increased with the length of the chewing cycle. In the T-limited condition, in which the subject did not determine the timing of swallowing, the chewing speed and number of cycles increased. This resulted in a decrease in Mas activity per cycle.

**Table 1:** Effect of experimental condition on chewing behavior.

	Free	N-limited	T-limited	P
N of cycles	24.9 ± 10.2	25.0 ± 10.1	27.1 ± 10.3*	<0.001
Chewing cycle time (sec)	0.64 ± 0.12	0.67 ± 0.12*	0.61 ± 0.09*	<0.001
Mas activity (mV·sec)	0.211 [0.092, 0.602]	0.212* [0.107, 0.656]	0.195 [0.076, 0.626]	0.01
Mas activity/cycle (mV·sec)	0.008 [0.002, 0.046]	0.009* [0.002, 0.051]	0.009*[0.002, 0.042]	<0.001
Supra activity (mV·sec)	0.135 [0.070, 0.337]	0.126 [0.075, 0.370]	0.147 [0.070, 0.384]	0.754
Supra activity/cycle (mV·sec)	0.006 [0.002, 0.021]	0.006* [0.002, 0.018]	0.006* [0.002, 0.018]	0.002

Values represent mean ± SD for N of cycles and chewing cycle time and median [IQR25%, 75%] for muscle activity. *P < 0.05 vs Free.

### Effect of experimental conditions on temporal qualities of chewing

To examine how the experimental conditions in the N-limited and T-limited tests affected the temporal qualities of chewing behavior, we compared chewing cycle time as well as Mas and Supra EMG activity per chewing cycle among the chewing sub-stages (early, middle, and late) and different conditions. Regarding the chewing cycle time, we found a statistically significant interaction between the sub-stage and test condition (*P* = 0.036). We observed significant differences in the chewing cycle time in the late stage between the free chewing and N-limited tests, in the late stage between the N and T-limited tests, and between the early and late and the middle and late stages in the N-limited test (Table 2).

**Table 2:** Effect of experimental condition on temporal changes in chewing behavior.

		Early	Middle	Late
Cycle time (sec)	Free	0.62 ± 0.12	0.59 ± 0.11	0.60 ± 0.10^d^
	N limited	0.63 ± 0.10^c^	0.61 ± 0.10^b^	0.66 ± 0.14
	T limited	0.59 ± 0.08	0.56 ± 0.08	0.58 ± 0.09^e^
		**Early** ^**a**^	**Middle** ^**a**^	**Late**
Mas activity per cycle (mV·sec)	Free	0.011 [0.003, 0.046]	0.010 [0.002, 0.048]	0.007 [0.001, 0.045]
	N limited^g^	0.011 [0.003, 0.054]	0.010 [0.002, 0.057]	0.007 [0.002, 0.042]
	T limited	0.009 [0.002, 0.040]	0.009 [0.002, 0.043]	0.007 [0.001, 0.042]
		**Early** ^**a**^	**Middle** ^**a**^	**Late**
Supra activity per cycle (mV·sec)	Free^f^	0.007 [0.002, 0.020]	0.006 [0.002, 0.031]	0.005 [0.002, 0.013]
	N limited	0.008 [0.002, 0.020]	0.006 [0.002, 0.018]	0.005 [0.001, 0.014]
	T limited	0.007 [0.002, 0.019]	0.006 [0.002, 0.019]	0.004 [0.002, 0.015]

Values represent mean ± SD for chewing cycle time and median [IQR25%, 75%] for muscle activity. ^a^P < 0.001 vs Late, ^b^P = 0.002 vs Late, ^c^P = 0.023 vs Late, ^d^P = 0.002 vs N-limited, ^e^P < 0.001 vs N-limited, ^f^P = 0.005 vs T-limited, ^g^P = 0.025 vs T-limited.

We found no statistically significant interactions between the sub-stage and test with respect to Mas or Supra activity per chewing cycle. In both muscles, we found a significant difference between activity in the early and late stages and that between the middle and late stages (Table 2). In addition, we found a significant difference in Mas activity per chewing cycle between the N- and T-limited tests and that in Supra activity per chewing cycle between the free chewing and T-limited tests.

### Effect of experimental condition on the transition from chewing to swallowing

**Figure 2:**
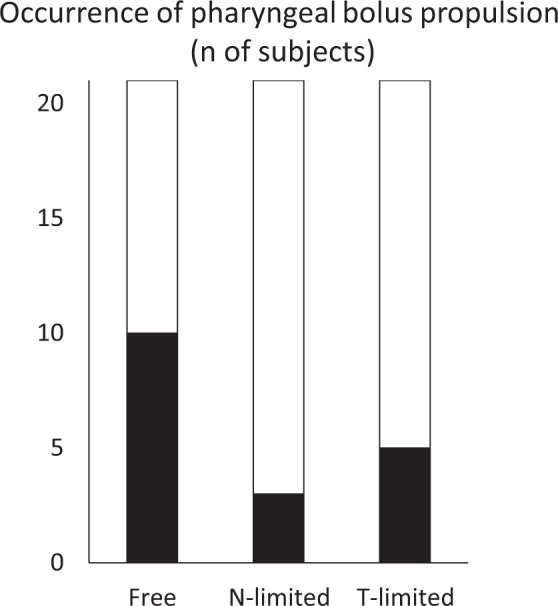
Effect of experimental condition on pharyngeal bolus propulsion. The number of participants who exhibited pharyngeal bolus propulsion was 10 in the free chewing test, 3 in the N-limited test, and 5 in the T-limited test (*P* = 0.02).

During the free chewing test, the bolus was often visible in the pharynx before swallowing. This is called Stage II transport^20^. Because Stage II transport was originally defined using videofluorographic (VF) images, we defined bolus transport into the pharynx during chewing to be “pharyngeal bolus propulsion” in the present study. Pharyngeal bolus propulsion was rare in the N- and T-limited tests (Fig. 1). The occurrence frequency of pharyngeal bolus propulsion in the free chewing test was much higher than that in the N- and T-limited tests (Fig. 2).

**Figure 3:**
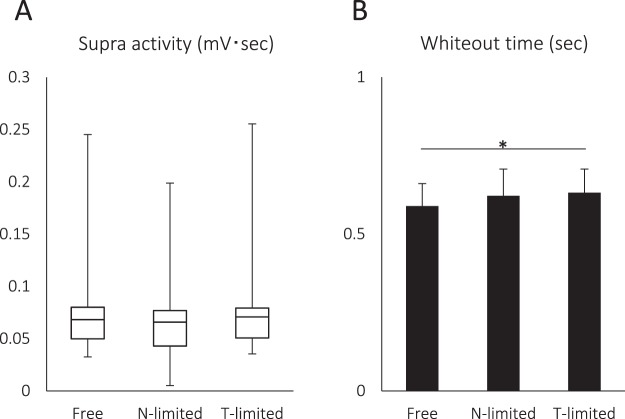
Effect of experimental condition on swallowing behavior. The mean swallowing-related Supra EMG activity was 0.065 [0.032–0.245] mV·sec (median [IQR25–75%]) in the free chewing test, 0.065 [0.005–0.198] mV·sec (median [IQR25–75%]) in the N-limited test, and 0.071 [0.035–0.255] mV·sec (median [IQR25–75%]) in the T-limited test. There was no significant difference among the conditions (*P* = 0.538). (**B**) The whiteout time was 0.59 ± 0.07 sec (mean ± SD) in the free chewing test, 0.62 ± 0.08 sec (mean ± SD) in the N-limited test, and 0.63 ± 0.08 sec (mean ± SD) in the T-limited test. There was a statistically significant difference among the conditions (*P* = 0.022). The whiteout time was significantly longer in the T-limited test compared with that in the free chewing test. *P = 0.023.

Although we observed no significant differences in Supra EMG activity among the tests, the whiteout time differed among the tests such that it was significantly longer in the T-limited test than in the free chewing test (Fig. 3). Finally, we compared the correlation of swallow-related activity in the right and left Supra EMG between trials with and without pharyngeal bolus propulsion. We found a significant positive correlation (*P* < 0.001) in both cases, but the correlation coefficient was much higher in trials with stage II transport (R^2^ = 0.6383 without pharyngeal bolus propulsion vs R^2^ = 0.9568 with pharyngeal bolus propulsion).

## Discussion

In the present study, we evaluated the movement of the bolus in the oral and pharyngeal cavities using VE, which is different than VF imaging. Abe *et al*. used VE imaging to observe the transport of the bolus from the mouth to the pharynx during chewing^21^. Although they discussed a limitation of endoscopic studies, namely that measurements of area are not precisely equal to those of volume, they concluded that assessing masticatory function via VE can produce a comprehensive evaluation of bolus transport during chewing^21,22^.

We used 8 g of steamed rice as test food because steamed rice is one of the most common foods in Japan and has been extensively used to evaluate masticatory and swallowing function in our previous studies^5,17,23^. When Wintergerest *et al*.^24^ investigated the within-subject variability of chewing performance, they used gums of different sizes and textures to evaluate within-subject variability. They concluded that a 2 g bolus of soft gum optimally reduced within-subject variability, increasing statistical power. However, there are marked differences in physical properties between gum and steamed rice. Gum has a firm texture and is not scattered during chewing, while steamed rice is readily broken down because it is relatively soft and also digestible via saliva, which contains amylase. Given these differences, we expected that 8 g might be a suitable amount of rice for subjects to chew comfortably and stably. Previous studies that investigated rice chewing used 5–11.5 g of steamed rice^17,25,26^. In the present study, all of the participants freely ate the rice in a natural manner and did not report any discomfort. We found no significant differences in the parameters evaluated among the three free chewing trials.

Recently, several new examinations for evaluating masticatory performance have been developed^27–29^. In these tests, subjects were asked to chew a gummy-jelly or chewing gum for a fixed number of strokes^27,28^ or in a fixed amount of time^29^, cued by the experimenter. Although these studies did not examine how chewing behavior was affected by an experimental manipulation, asking subjects to concentrate on chewing specific materials might impact chewing rhythm among other factors. Because the subjects were allowed to swallow the bolus following chewing in the N-limited test in the present study, we predicted that bolus formation and propulsion would not vary from those in the free chewing test (see Results).

In the T-limited test, the subjects were asked to swallow when they received a cue from the experimenter. They were allowed to chew the food freely. Palmer *et al*.^18^ recorded volitional chewing behavior that occurred when subjects were asked to chew a cookie until they were ready to swallow, and then swallow when they received a cue from the experimenter. This method was similar to that used in the T-limited test in the present study in that the subject did not swallow without receiving the experimenter cue. Although we predicted that chewing behavior would not differ between the free chewing test and the T-limited test because the subjects were allowed to chew freely, we found that chewing cycle time in the T-limited test was shorter and Mas and Supra activity were smaller compared with those in the free chewing test. Palmer *et al*.^18^ found that chewing duration was longer with vs without a command. This was primarily due to an increase in the number of chewing cycles (although the authors did not measure the chewing rhythm). The power stroke generated by the Mas might have been weaker in the T-limited condition compared with that in the free chewing test.

In the present study, we divided chewing performance prior to the first swallow into three stages, as described in previous studies^5,11,30^. As we expected, in the free chewing test, Mas activity per cycle gradually decreased, and significantly decreased at the late stage of chewing. Chewing performance has been found to depend largely on the hardness of foods, and chewing behavior adapts to changes in the hardness of the bolus during chewing^5,11,31–33^. When chewing rice, the hardness of the rice changes in a time-dependent way, specifically, hardness gradually decreases until swallowing. Other parameters, such as the adhesiveness and cohesiveness of rice, undergo less change^5^. In contrast to the results of the present study, our previous report showed that Mas activity per chewing cycle did not change throughout the masticatory sequence^5^. This contradiction may be due to the difference in the volume of the food stimulus in the two studies; 8 g in the present study vs 15 g in our previous study. The difference in volume may also have affected chewing performance^34–36^. In fact, the cycle time was different between these studies. In the study with 15 g of rice, the cycle was 0.77 sec in the early stage, 0.68 sec in the middle stage, and 0.66 sec in the late stage. In the study with 8 g of rice, the cycle was 0.62 sec in the early stage, 0.59 sec in the middle stage, and 0.60 sec in the late stage.

We also found that Supra EMG activity per chewing cycle gradually decreased. The basic pattern of Supra activity is generally determined by the size of the food object^25^ and related to the gape of the jaw^37^. In the present study, the chewing cycle time did not change with the reduction in Supra EMG activity. A previous study reported that the masticatory rhythm is mainly controlled by the activity pattern of jaw-opening muscles such as the Supra muscles^38^. Although we did not conduct a cycle-by-cycle evaluation of the relationship between changes in Supra EMG activity and chewing cycle time, the volume and texture of food may affect temporal changes in chewing behaviors. Although we did not use a large volume of food in the present study, the physical properties of the bolus might have affected the activity of jaw-opening muscles. As the introduction of food to the mouth is expected to require a large jaw-opening movement at the beginning of chewing, Supra EMG activity may depend not only on food hardness but also on the size of the food stimulus.

In the present study, we found that the Mas activity per chewing cycle in the late stage was significantly larger in the N-limited test vs the T-limited test, while Supra activity per chewing cycle was significantly larger in the free chewing test vs the T-limited test. In addition, pharyngeal bolus propulsion occurred less frequently in the N- and T-limited tests compared with the free chewing test. Palmer *et al*.^20^ collected VF images and found that transport of the food bolus when chewing solid foods was actively driven by tongue-palate contact and did not depend on gravity. Saitoh *et al*.^39^ used solid foods and liquids to examine the effect of chewing on the relationship between bolus transport and swallow initiation. The authors found that, in most cases, including conditions with liquids, chewing movements resulted in transport of the bolus into the hypopharynx before swallowing initiation. Hori *et al*.^40^ recorded tongue pressure against the palate as well as jaw movement during mastication in humans, and found that when chewing solid food, the magnitude of force and duration of time during which the tongue was pressed against the hard palate were significantly greater during the late stage vs early stage of chewing. During chewing, food breakdown, bolus formation, and bolus propulsion are necessary for swallowing. These data suggest that masticatory function adapts throughout the process of bolus formation towards the swallowing event. Palmer *et al*.^18^ demonstrated that volitional chewing drastically changed bolus transport in the oral and pharyngeal cavities, leading to a significant delay. Thus, higher centers involved in chewing, such as the cerebral cortex, appear to modulate orofacial movements and therefore bolus transport prior to swallowing. In other words, chewing and swallowing movements may be consciously altered by volition.

We hypothesized that bolus transport into the pharynx prior to swallowing would be related to measures of swallowing performance such as Supra activity and/or whiteout time, which represents the duration of pharyngeal swallowing^41^. As a result, whiteout time in the T-limited test was longer than that in the free chewing test. In our study, subjects were free to chew the food in all tests, so we predicted that the physical properties of the food bolus would be changed in a similar manner among the conditions. If swallowing performance differed among the tests, we would expect this difference to be attributable to differences in the bolus properties and/or bolus location at the start of pharyngeal swallowing. Furuya *et al*.^19^ showed that chewing with a conscious effort increased stage II transport time and reduced bolus transit time during pharyngeal swallowing. They concluded that volitional chewing with a conscious effort can alter bolus transport and swallowing, resulting in easier swallowing. This suggests that the location of the bolus may affect pharyngeal swallowing. However, this is in contrast with the finding that the whiteout time in the N-limited test was unaffected by the reduction in the occurrence of bolus propulsion during chewing.

In the T-limited test, compared with the free chewing test, the chewing cycle time was shorter while Mas and Supra activities were smaller. Hard and solid foods are known to require large^4,42,43^ and long^44,45^ jaw-closing muscle activities (such as those involving the Mas), which can elongate chewing cycle time. If the food stimulus in this study had been harder, masticatory efficacy might have been lower in the T-limited condition, giving the bolus different properties compared with those in the free chewing test. Our data support this theory; Mas and Supra activity per cycle at the late stage were significantly smaller in the T-limited test than those in either the N-limited test or the free chewing test. When participants were instructed not to swallow, chewing behavior prior to swallowing was gradually affected.

Several limitations affect the interpretation of the present findings. First, we recruited only 21 healthy young participants: 11 men and 10 women. Although no participants reported any discomfort or fatigue when eating 8 g of steamed rice repeatedly, this may not be the case for participants of all ages^46^. Second, we could not identify the muscles that contributed to the formation and transport of the bolus during chewing, i.e., those implicated in chewing and swallowing performance. Third, our data may have been impacted by the order effect. In the present study, the N- and T-limited tests were always carried out following the free chewing test. However, a more comprehensive evaluation of how repeated measurements and order affected the results would have been helpful. Fourth, we might have underestimated the effect of salivation. While chewing solid food, both the reduction of the food structure and lubrication of the bolus are necessary to move the bolus smoothly. Iguchi *et al*.^5^ reported a positive correlation between the stimulated salivary flow rate and change in Mas activity during chewing. Thus, salivary flow may affect individual chewing processes. Further research should examine in detail how the muscles involved in chewing and swallowing are affected not only by attention but also salivation, as well as the influence of age on the entire masticatory sequence.

## Materials and Methods

### Participants

Twenty-one healthy volunteers (11 men, 10 women), ranging in age from 21 to 41 years (average age ± SD, 26.0 ± 4.7 years) participated in this study. Prior to starting the study, a dentist confirmed that the participants had no abnormalities in the number or position of their teeth, temporomandibular disorders, occlusal abnormalities, mastication problems, or swallowing problems. No participant had a history of alimentary, pulmonary, or neurological disease, structural or speech disorders, or voice problems. Informed consent was obtained from all participants after receiving information about the experimental procedure, and this study was approved by the Ethics Committee of the Niigata University Graduate School of Medical and Dental Sciences (23-R16-11-08). The experiments were performed in accordance with the Declaration of Helsinki (2008) for humans.

### EMGs and VE images

Surface EMGs were recorded from the Mas (jaw-closer) and Supra (jaw-opener and hyoid-elevator) muscles on the right and left sides of the submental region. Electrodes (ZB-150H; Nihon Kohden, Tokyo, Japan) were attached to the skin over the center of the Mas muscle and the anterior belly of the digastric muscle (one of the Supra muscles) with an inter-electrode distance of 2 cm. Signals were filtered and amplified (high pass, 30 Hz to remove movement-related artifacts and low pass, 2 kHz) (WEB-1000; Nihon Kohden). Flexible endoscopy was performed to observe bolus transport in the pharynx. A fiber-optic endoscope (FNL-10RP3; PENTAX, Tokyo, Japan) was inserted through the nasal passage and into the midpharynx. All signals were collected via an interface board (PowerLab; ADInstruments, Colorado Springs, CO, USA) and stored on a personal computer. The sampling rate was 10 kHz for EMGs and 33 Hz for VE images. Data synchronization and analysis were performed using the PowerLab software package (Video Module and LabChart6; ADInstruments), which automatically aligned the data at different sampling rates.

### Data collection

#### Test food

In the present study, Japanese steamed rice was prepared as a test food. Steamed rice is a traditional staple food in Japan, and has been used extensively for the evaluation of masticatory and swallowing functions^5,23,25,26,47–49^. We confirmed that all the participants in the present study were Japanese and enjoyed eating steamed rice. The rice was kept at around 36 degrees Celsius in the rice cooker until just before it was offered to the participants.

#### Ingestion recording (free chewing test)

The participants were asked not to eat or drink for at least 1 h prior to each experiment. Individual subjects were instructed to sit comfortably in a chair with head support. According to our previous method, we determined that 8 g was an appropriate amount of rice for use in each trial in the present study^17^.

During the experiment, the food samples, which were roughly hemispherical in shape, were placed on a dish in front of the participants. First, the participants were asked to take and freely eat a food sample using a spoon, and to repeat this action twice for a total of three times. In the masticatory sequence, most food is swallowed during the first swallow, and any residual food is aggregated by the tongue into a bolus and then swallowed in the last swallow^23,26^. Thus, the process of bolus formation is expected to occur before the first swallow. Accordingly, we measured the chewing duration up to initiation of the first swallow, as follows. The Mas and Supra EMG bursts were first full-wave rectified and smoothed (time constant 20 msec). The onset and offset thresholds were defined according to a previous method^5,50^. Specifically, EMGs recorded at rest were rectified for 5 sec and their mean values ± SD were obtained as controls. When the trial values exceeded the control values + 3SDs, EMG bursts were considered to indicate muscle activity. We calculated the chewing duration, defined as the time between the onset of the Mas EMG burst in the first chewing cycle and the onset of the Mas EMG burst prior to swallowing, and the number of chewing cycles. We used the average number of chewing cycles (N) and the average chewing duration (T) from the three trials for the following tests in each individual.

#### Number limited and time limited chewing tests

Participants took part in two additional tests: the N- and T-limited tests. In the former, the participants were asked to complete a set number of chewing cycles before executing their first swallow (N). They were asked to chew the food in a natural manner while counting the number of chewing cycles by themselves, and to swallow the food when they reached N. In the latter, the participants were instructed to chew the food in a natural manner for T sec and swallow it when the experimenter gave a cue. The participants were blinded to the passage of time throughout the recording sessions. The time interval between the trials was at least 2 min, and subjects were able to rinse their mouths with distilled water whenever they wished between the trials. The order of presentation of the N- and T-limited tests was randomly determined.

### Data analysis

#### Differences in chewing behavior

We first investigated how the experimental conditions, i.e., the N- and T-limited tests, affected overall chewing behaviors. For each trial, we used filtered EMGs to measure the number of chewing cycles until the first swallow and the chewing cycle time. As with those values, we calculated Mas and Supra activities, including areas containing filtered EMG bursts, until the first swallow and per chewing cycle. The chewing cycle time was defined as the total chewing duration divided by the number of chewing cycles, i.e., the time between the onsets of two consecutive Mas EMG bursts on the right side during chewing. As the chewing side could not easily be detected from the recordings, the EMG bursts for the right and left sides were averaged. The mean values were compared among the tests (free, N-limited, and T-limited) using a one-way repeated measures analysis of variance on ranks followed by Dunnett’s test. Regarding the data obtained from the free chewing test, we utilized the average value of the three trials.

Next, we evaluated temporal changes in muscle activity. For this purpose, the chewing duration in each trial was divided into three periods or substages: early (first third), middle (second third), and late (last third) according to the number of chewing cycles for each individual. We ascertained the average chewing cycle time and EMG activity per chewing cycle for each sub-stage, and the mean values were compared among the tests and sub-stages using a two-way repeated measures analysis of variance (only for the cycle time) or a two-way repeated measures analysis of variance on ranks followed by Tukey’s HSD test.

#### Differences in the transition from chewing to swallowing

When chewing solid foods, a triturated bolus is often propelled into the pharynx via a process termed ‘pharyngeal bolus propulsion’ before swallowing. We compared the number of subjects who exhibited pharyngeal bolus propulsion among the conditions using a chi-square test. The start of pharyngeal bolus propulsion and onset and offset of pharyngeal swallowing (i.e., whiteout) were determined via visual inspection of VE images^41^.

Finally, we examined the effects of experimental condition on swallowing behavior. We compared swallow-related Supra EMG activity and whiteout time (defined as the time interval between the onset and offset of whiteout) among the conditions using a one-way repeated measures analysis of variance on ranks and a one-way repeated measures analysis of variance followed by Tukey’s HSD test, respectively.

Statistical analyses were performed using Sigmaplot software (Sigmaplot 12.0, Systat Software Inc., CA, USA). P values < 0.05 were considered significant. All values are expressed as means ± SD.

## References

[1] Lund JP, Kolta A, Westberg KG, Scott G (1998). Brainstem mechanisms underlying feeding behaviors. Curr Opin Neurobiol.

[2] Curby WA (1953). A simplified instrument for measurement of food platform area. J Dent Res.

[3] Gibbs CH (1981). Occlusal forces during chewing–influences of biting strength and food consistency. J Prosthet Dent.

[4] Horio T, Kawamura Y (1989). Effects of texture of food on chewing patterns in the human subject. J Oral Rehabil.

[5] Iguchi H (2015). Changes in jaw muscle activity and the physical properties of foods with different textures during chewing behaviors. Physiol Behav.

[6] Yurkstas A (1953). The effect of masticatory exercise on the maximum force tolerance of individual teeth. J Dent Res.

[7] Proschel P, Hofmann M (1988). Frontal chewing patterns of the incisor point and their dependence on resistance of food and type of occlusion. J Prosthet Dent.

[8] Thexton AJ, Hiiemae KM, Crompton AW (1980). Food consistency and bite size as regulators of jaw movement during feeding in the cat. J Neurophysiol.

[9] Luschei ES, Goodwin GM (1974). Patterns of mandibular movement and jaw muscle activity during mastication in the monkey. J Neurophysiol.

[10] Pierson A, Magnen JL (1970). Study of Food Textures By Recording of Chewing and Swallowing Movements. J Texture Stud.

[11] Hiiemae K (1996). Natural bites, food consistency and feeding behaviour in man. Arch Oral Biol.

[12] Mathevon E, Mioche L, Brown WE, Culioli J (1995). Texture analysis of beef cooked at various temperatures by mechanical measurements, sensory assessments and electromyography. J Texture Studies.

[13] Mioche L, Martin JF (1998). Training and sensory ijdgement effects on mastication as studied by electromyography. J Food Science.

[14] Lin CS (2017). Age- and sex-related differences in masseter size and its role in oral functions. J Am Dent Assoc.

[15] Mioche L, Bourdiol P, Martin JF, Noel Y (1999). Variations in human masseter and temporalis muscle activity related to food texture during free and side-imposed mastication. Arch Oral Biol.

[16] Woda A, Foster K, Mishellany A, Peyron MA (2006). Adaptation of healthy mastication to factors pertaining to the individual or to the food. Physiol Behav.

[17] Iizumi T, Magara J, Tsujimura T, Inoue M (2017). Effect of body posture on chewing behaviours in healthy volunteers. J Oral Rehabil.

[18] Palmer JB, Hiiemae KM, Matsuo K, Haishima H (2007). Volitional control of food transport and bolus formation during feeding. Physiol Behav.

[19] Furuya J, Hara A, Nomura T, Kondo H (2014). Volitional chewing with a conscious effort alters and facilitates swallowing during feeding sequence. J Oral Rehabil.

[20] Palmer JB (1998). Bolus aggregation in the oropharynx does not depend on gravity. Arch Phys Med Rehabil.

[21] Abe R, Furuya J, Suzuki T (2011). Videoendoscopic measurement of food bolus formation for quantitative evaluation of masticatory function. J Prosthodont Res.

[22] Dua KS, Ren J, Bardan E, Xie P, Shaker R (1997). Coordination of deglutitive glottal function and pharyngeal bolus transit during normal eating. Gastroenterology.

[23] Shiozawa M (2012). Differences in chewing behavior during mastication of foods with different textures. J Texture Studies.

[24] Wintergerst AM, Throckmorton GS, Buschang PH (2008). Effects of bolus size and hardness on within-subject variability of chewing cycle kinematics. Arch Oral Biol.

[25] Kohyama K (2007). Textural evaluation of rice cake by chewing and swallowing measurements on human subjects. Biosci Biotechnol Biochem.

[26] Okada A, Honma M, Nomura S, Yamada Y (2007). Oral behavior from food intake until terminal swallow. Physiol Behav.

[27] Hama Y, Kanazawa M, Minakuchi S, Uchida T, Sasaki Y (2014). Properties of a color-changeable chewing gum used to evaluate masticatory performance. J Prosthodont Res.

[28] Okiyama S, Ikebe K, Nokubi T (2003). Association between masticatory performance and maximal occlusal force in young men. J Oral Rehabil.

[29] Shiga H, Kobayashi Y, Katsuyama H, Yokoyama M, Arakawa I (2012). Gender difference in masticatory performance in dentate adults. J Prosthodont Res.

[30] Kohyama K, Sasaki T, Hayakawa F (2008). Characterization of food physical properties by the mastication parameters measured by electromyography of the jaw-closing muscles and mandibular kinematics in young adults. Biosci Biotechnol Biochem.

[31] Foster KD, Woda A, Peyron MA (2006). Effect of texture of plastic and elastic model foods on the parameters of mastication. J Neurophysiol.

[32] Lassauzay C, Peyron MA, Albuisson E, Dransfield E, Woda A (2000). Variability of the masticatory process during chewing of elastic model foods. Eur J Oral Sci.

[33] Peyron, A., Lassauzay, C. & Woda, A. Effects of increased hardness on jaw movement and muscle activity during chewing of visco-elastic model foods. *Exp Brain Res* **142**, 41–51. Epub 2001 Nov 2009 (2002). 10.1007/s00221-001-0916-5 11797083

[34] Diaz-Tay J, Jayasinghe N, Lucas PW, McCallum JC, Jones JT (1991). Association between surface electromyography of human jaw-closing muscle and quantified food breakdown. Arch Oral Biol.

[35] Lucas PW, Ow RK, Ritchie GM, Chew CL, Keng SB (1986). Relationship between jaw movement and food breakdown in human mastication. J Dent Res.

[36] Ottenhoff FA, van der Bilt A, van der Glas HW, Bosman F (1993). Control of human jaw elevator muscle activity during simulated chewing with varying bolus size. Exp Brain Res.

[37] Meng Y, Uchida K, Sato T, Yamamura K, Yamada Y (1999). Difference in the burst patterns of digastric and mylohyoid activities during feeding in the freely behaving rabbit. Dysphagia.

[38] Lund JP (1991). Mastication and its control by the brain stem. Crit Rev Oral Biol Med.

[39] Saitoh E (2007). Chewing and food consistency: effects on bolus transport and swallow initiation. Dysphagia.

[40] Hori K, Ono T, Nokubi T (2006). Coordination of tongue pressure and jaw movement in mastication. J Dent Res.

[41] Shiino Y (2016). Effect of body posture on involuntary swallow in healthy volunteers. Physiol Behav.

[42] Hidaka O (1997). Regulation of masticatory force during cortically induced rhythmic jaw movements in the anesthetized rabbit. J Neurophysiol.

[43] Mioche L, Peyron MA (1995). Bite force displayed during assessment of hardness in various texture contexts. Arch Oral Biol.

[44] Kakizaki Y, Uchida K, Yamamura K, Yamada Y (2002). Coordination between the masticatory and tongue muscles as seen with different foods in consistency and in reflex activities during natural chewing. Brain Res.

[45] Plesh O, Bishop B, McCall W (1986). Effect of gum hardness on chewing pattern. Exp Neurol.

[46] Peyron MA, Mishellany A, Woda A (2004). Particle size distribution of food boluses after mastication of six natural foods. J Dent Res.

[47] Iida Y, Katsumata A, Fujishita M (2011). Videofluorographic evaluation of mastication and swallowing of Japanese udon noodles and white rice. Dysphagia.

[48] Kohyama K (2005). Mastication effort estimated by electromyography for cooked rice of differing water content. Biosci Biotechnol Biochem.

[49] Tada H, Kaseno K, Naito S, Oshima S (2007). Non-contact three-dimensional mapping and ablation of swallowing-induced atrial tachyarrhythmias: two case reports. J Cardiovasc Electrophysiol.

[50] Tsukada T, Taniguchi H, Ootaki S, Yamada Y, Inoue M (2009). Effects of food texture and head posture on oropharyngeal swallowing. J Appl Physiol.

